# Acyclic retinoid and angiotensin-II receptor blocker exert a combined protective effect against diethylnitrosamine-induced hepatocarcinogenesis in diabetic OLETF rats

**DOI:** 10.1186/s12885-018-5099-6

**Published:** 2018-11-26

**Authors:** Norihisa Nishimura, Kosuke Kaji, Mitsuteru Kitade, Yosuke Aihara, Shinya Sato, Kenichiro Seki, Yasuhiko Sawada, Hiroaki Takaya, Yasushi Okura, Hideto Kawaratani, Kei Moriya, Tadashi Namisaki, Akira Mitoro, Hitoshi Yoshiji

**Affiliations:** Third Department of Internal Medicine, Nara Medical University, 840 Shijo-cho, Kashihara, Nara 634-8522 Japan

**Keywords:** Acyclic retinoid, ARB, Hepatocarcinogenesis, Chemoprevention, Insulin resistance

## Abstract

**Background:**

Insulin resistance (IR) is closely associated with the progression of hepatocellular carcinoma (HCC). Acyclic retinoid (ACR) targets retinoid X receptor α and reportedly prevents HCC recurrence in clinical practice. Angiotensin-II receptor blocker (ARB) can also inhibit experimental hepatocarcinogenesis and HCC development. These are reported to suppress IR-based hepatocarcinogenesis; however, limited data are available regarding the combined effects of both these agents. This study aimed to investigate the combined chemopreventive effect of ACR and ARB on liver tumorigenesis on rats with congenital diabetes.

**Methods:**

Male diabetic Otsuka Long-Evans Tokushima Fatty (OLETF) and non-diabetic Long-Evans Tokushima Otsuka (LETO) rats underwent 70% partial hepatectomy following a single intraperitoneal injection of diethylnitrosamine to induce hepatocarcinogenesis and the administration of ACR (peretinoin, 40 mg/kg/day), ARB (losartan, 30 mg/kg/day), and a combination of ACR and ARB. Six weeks thereafter, we assessed the size and number of the pre-neoplastic lesions (PNL) as well as the altered angiogenesis, oxidative stress, and chronic inflammation in the liver. Moreover, we assessed the effects exerted by ACR and ARB on in vitro cell growth in human HCC cell lines and human umbilical vascular endothelial cells (HUVECs).

**Results:**

OLETF rats showed increase in the size and number of PNLs compared to LETO rats. ACR suppressed the augmentation in size and number of PNLs in the OLETF rats with suppression of cell growth, intrahepatic angiogenesis, lipid peroxidation, oxidative DNA damage, and proinflammatory cytokine production. Combining ACR with ARB enhanced the tumor-suppressive effect and ameliorated intrahepatic angiogenesis, lipid peroxidation, and proinflammatory status; however, cell growth and oxidative DNA damage remained unchanged. IR-mimetic condition accelerated in vitro proliferative activity in human HCC cells, while ACR inhibited this proliferation with G0/G1 arrest and apoptosis. Furthermore, ACR and ARB significantly attenuated the HUVECs proliferation and tubular formation under the IR-mimetic condition, and a combination of both agents demonstrated greater inhibitory effects on HUVEC growth than each single treatment.

**Conclusions:**

ACR and ARB exert a combined inhibitory effect against IR-based hepatocarcinogenesis by the inhibition of cell growth, intrahepatic angiogenesis, and oxidative stress. Thus, this combination therapy appears to hold potential as a chemopreventive treatment therapy against HCC.

**Electronic supplementary material:**

The online version of this article (10.1186/s12885-018-5099-6) contains supplementary material, which is available to authorized users.

## Background

Hepatocellular carcinoma (HCC) is the fifth most common cancer and the third leading cause of cancer-related deaths worldwide [1]. HCC prognosis is generally poor owing to the high recurrence rate; 50% of HCC patients experience recurrence following curative treatment, including surgical resection and ablation [2, 3]. Therefore, chemoprevention for either primary or secondary HCC development can be beneficial for improving prognosis.

Insulin resistance (IR) commonly develops in chronic liver diseases, including liver cirrhosis. Further, recent studies have demonstrated that Type 2 diabetes mellitus (T2DM) with IR is one of the most powerful risk factors for HCC progression [3–6]. T2DM is associated with a two- to three-fold increased risk of HCC, irrespective of the etiology [7, 8]. In fact, basic experiments that have used congenitally diabetic rats revealed that the IR status, defined by the coexistence of high blood glucose and high insulin levels, directly accelerated diethylnitrosamine (DEN)-induced hepatocarcinogenesis [9]. Moreover, recent epidemiological studies have shown an increased proportion of non-B/non-C HCC in keeping with the rising prevalence of non-alcoholic fatty liver diseases (NAFLD) during the previous decade [10, 11]. NAFLD is generally associated with IR and includes the spectrum of simple steatosis and non-alcoholic steatohepatitis (NASH), recognized as a potentially progressive disease that may lead to HCC. These basic and clinical evidences point to the strong need for new chemopreventive strategies against IR-based hepatocarcinogenesis.

Acyclic retinoid (ACR, NIK-333) was developed in Japan as an oral synthetic retinoid that targets retinoid nuclear receptors, including retinoid X receptor (RXR) and retinoic acid receptor (RAR) [12]. Several clinical trials have demonstrated significant reduction in the post-therapeutic recurrence of HCC and improved survival rate in patients who were administered ACR [13, 14]. In addition, ACR reportedly exerts chemopreventive effects on hepatocarcinogenesis in some rodent models [15–17]. ACR leads to growth suppression in human HCC cells by inducing cell cycle arrest at G0/G1 phase and apoptosis. It is noteworthy that as per recent basic research, ACR treatment can prevent obesity-related liver tumorigenesis and alleviate IR, liver steatosis, and inflammation [18]. Thus, ACR is expected to be a novel agent for reducing the HCC incidence in patients with liver cirrhosis. In fact, larger-scale clinical trials are currently ongoing to validate ACR efficacy in preventing HCC development; however, no satisfactory outcomes have been reported. This indicates that ACR monotherapy may not exert sufficiently strong inhibitory effects to enable HCC chemoprevention. Therefore, we postulated that the combination therapy of ACR and another agent with more potent anticancer effects, especially on IR-based hepatocarcinogenesis, would be more effective.

Angiotensin-converting enzyme inhibitor (ACE-I) and angiotensin-II type1 receptor blocker (ARB), key drugs for renin-angiotensin-aldosterone system (RAAS) blockade, are widely used in clinical practice for managing arterial hypertension, heart failure, myocardial infarction, and chronic kidney disease without any serious adverse effects. It is interesting to note that as per several researches, ACE-I and ARB possess strong antiangiogenic activity, and these agents could inhibit tumor growth in several types of cancers, including HCC using clinically comparable doses [19, 20]. ACE-I and ARB have also shown to subside IR in clinical practice [21]. We previously reported that in addition to inhibiting HCC growth, ACE-I/ARB significantly suppressed hepatocarcinogenesis even in the presence of IR along with the inhibition of vascular endothelial growth factor (VEGF) in the liver [22].

Here, we examined whether the administration of a combined therapy of ACR and ARB significantly inhibited the occurrence and development of IR-based hepatocarcinogenesis in rats with congenital diabetes. Furthermore, we analyzed the underlying mechanisms of the therapeutic effects of both agents in relation to cancer cell survival, angiogenesis, and oxidative stress.

## Methods

### Animals and regents

Eight-week-old male diabetic Otsuka Long-Evans Tokushima Fatty (OLETF) rats and littermate Long-Evans Tokushima Otsuka (LETO) rats were procured from the Otsuka Pharmaceutical Co. (Tokushima, Japan). The rats were housed in stainless steel mesh cages under controlled conditions (temperature: 23 °C ± 3 °C, relative humidity: 50% ± 20%, 10–15 air changes/h, illumination: 12 h/d). The rats were permitted ad libitum access to tap water throughout the study period. Peretinoin (NIK-333), as an ACR, was provided by the Kowa Company Ltd. (Nagoya, Japan), and losartan potassium, as an ARB, was purchased from Merck Ltd. (Tokyo, Japan).

### Experimental protocol

**Fig. 1:**
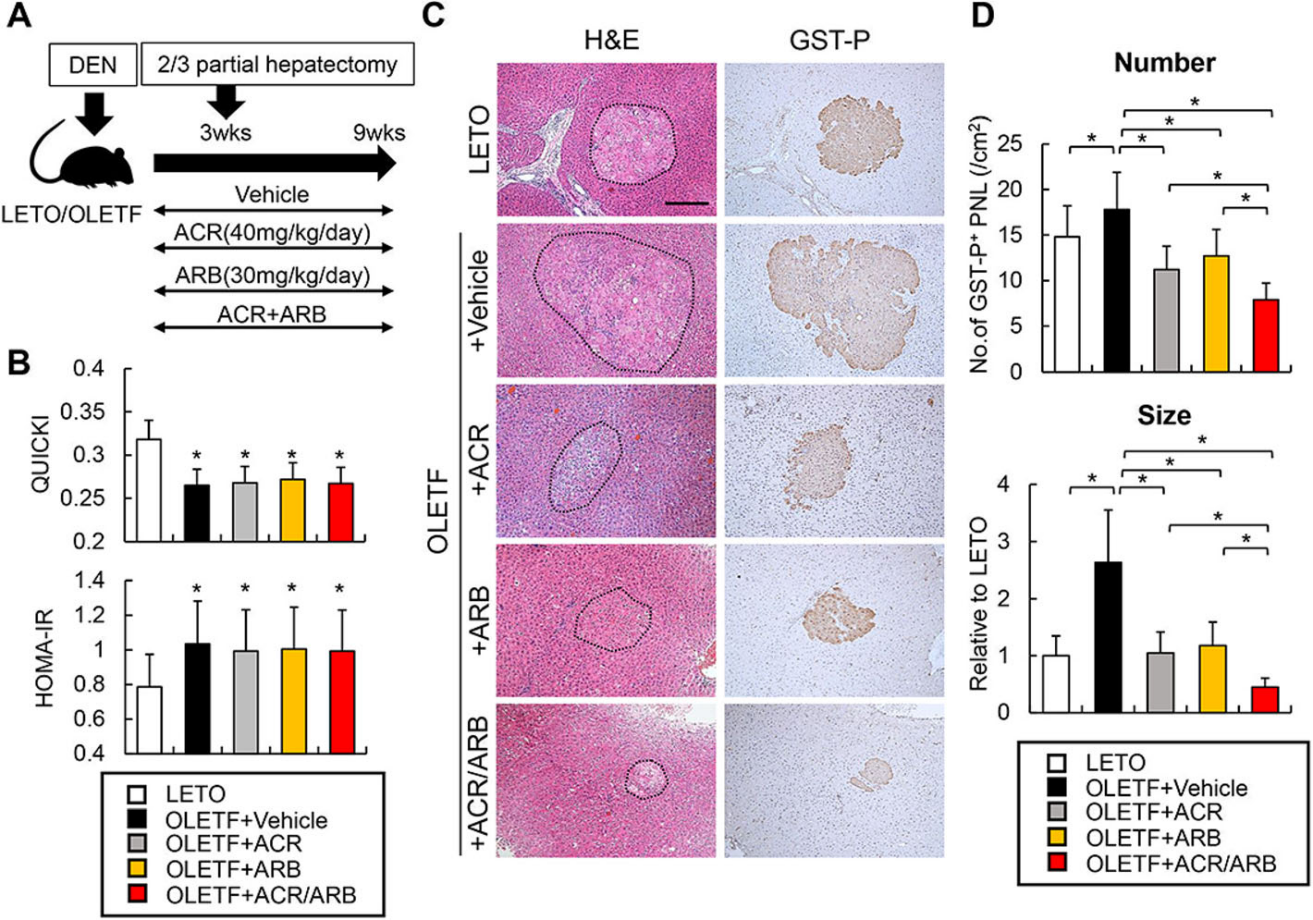
Effects of acyclic retinoid (ACR) and angiotensin-II receptor blocker (ARB) on hepatocarcinogenesis in diabetic rats. **a** Schematic of diethylnitrosamine (DEN)-induced rat hepatocarcinogenesis models. **b** The values of Quantitative Insulin Sensitivity Check Index (QUICKI) (Upper panel) and Homeostasis model assessment-Insulin Resistance (HOMA-IR) (Lower panel) in experimental rats. **c** Representative microphotographs of hematoxylin and eosin (H&E) staining (Left panels) and glutathione S-transferase (GST-P)-positive preneoplastic foci (Right panels). **d** Number of GST-P positive neoplastic lesions (GST-P^+^ PNL) per square centimeter (Upper panel) and relative size of GST-P^+^ PNL (Lower panel). LETO; Long-Evans Tokushima Otsuka rat, OLETF; Otsuka Long-Evans Tokushima Fatty rat. Data are mean ± SD (*n* = 10). *, *P* ≤ 0.01 as compared with the values of LETO (**b**); *, *P* ≤ 0.01 between each group (**d**)

The experimental design has been presented in Fig. 1a. The OLETF rats were administered intraperitoneal injection of 200 mg/kg diethylnitrosamine (DEN) (Tokyo Kasei Kogyo Co., Ltd., Tokyo, Japan) and were divided into the following four treatment groups (*n* = 10 each): vehicle (soybean oil), ACR (peretinoin, 40 mg/kg), ARB (losartan, 30 mg/kg), and ACR (40 mg/kg) + ARB (30 mg/kg), administered through daily oral gavage. As a negative control, LETO rats (n = 10) were also injected with DEN and treated with the vehicle. All experimental rats received 2/3 partially hepatectomies 3 weeks after the DEN injection for the promotion of hepatocarcinogenesis as described previously [9, 22]. All the rats were sacrificed 9 wk. after DEN injection. At the end of the experiment, the rats were anesthetized, their abdominal cavities were opened, blood samples were drawn via an aortic puncture, and livers were harvested for histological findings. Serum biological markers were assessed using routine laboratory methods. IR and insulin sensitivity were evaluated using the Homeostasis model assessment-Insulin Resistance (HOMA-IR) and the Quantitative Insulin Sensitivity Check Index (QUICKI), respectively, as described previously [9]. All the animal procedures were performed as per the recommendations of the Guide for Care and Use of Laboratory Animals (National Research Council). The study was approved by the animal facility committee of Nara Medical University.

### Intrahepatic triglyceride, 4-HNE, and 8-OHdG measurement

In order to determine the intrahepatic triglyceride content, the liver tissue homogenates were extracted using 2:1 (vol/vol) chloroform/methanol. Chloroform/methanol was added to the homogenate, and the mixture was then shaken for 15 min. Following centrifugation at 14,000 rpm for 10 min, the organic layer was collected. This extraction was repeated thrice. The collected sample was dried and resuspended in 1% Triton X-100/ethanol. The measurement was conducted using Triglyceride E-test Wako (Wako Pure Chemical Industries, Ltd., Osaka, Japan). The hepatic 4-Hydroxynonenal (4-HNE) content was measured using the 4 Hydroxynonenal ELISA Kit (MyBioSource, San Diego, CA, USA) as per the manufacturer’s protocol.

For determining the liver 8-hydroxy-2′-deoxyguanosine (8-OHdG) levels, DNA was extracted from the frozen liver tissue using a DNeasy mini kit (Qiagen, Tokyo, Japan) and purified as described previously [23]. The 8-OHdG level in the extracted DNA solution was determined using the highly sensitive 8-OHdG check ELISA kit (Nikken Seil Co., Ltd.). The 4-HNE and 8-OHdG levels were indicated as a ratio to those of the negative control group.

### Histological and immunohistochemical analyses

The liver sections were fixed with 10% formalin and embedded in paraffin. Then, 5-μm paraffin sections were stained with hematoxylin and eosin. For detecting the pre-neoplastic lesions, immunohistostaining was performed using anti-placental glutathione S-transferase (GST-P) polyclonal antibody (Medical & Biological Laboratories Co., Nagoya, Japan). Semi-quantitative analyses of the size of the GST-P positive pre-neoplastic lesions were conducted using the NIH ImageJ software (http://imagej.nih.gov/ij/).

### Cell culture and proliferation assay

Two human liver cancer cell lines Huh7 (JCRB0403) and HLE (JCRB0404) and human umbilical vascular endothelial cells (HUVECs) (IFO50271) were obtained from the Japanese Collection of Research Bioresources Cell Bank (Osaka, Japan). Cells were cultured in Dulbecco’s modified Eagle’s medium (Thermo Fisher Scientific K.K., Kanagawa, Japan) supplemented with 10% fetal bovine serum and 1% ampicillin/streptomycin.

Cell proliferation was assessed using water-soluble tetrazolium salt − 1 assay as per the manufacturer’s protocol. Initially, for the validation of the inducible effects of insulin on cell proliferation, the cells were treated with different dilutions of recombinant human insulin (rh-insulin) (0, 5, 10, 20, 30, and 40 μU/mL) (Gibco, Tokyo, Japan). Thereafter, we compared the effects of ACR (peretinoin, 10 μM), ARB (losartan, 10 μM), and the combined treatment (each 10 μM) on cell proliferation of Huh7, HLE, and HUVECs that were cultured under high concentrations of D-(+)-glucose (Nacalai Tesque, Kyoto, Japan) and rh-insulin. The cells were seeded on 96-well uncoated plastic dishes at a density of 5 × 10^4^ cells/mL, and each treatment was started 12 h after seeding and incubated for another 24 h. Three independent experiments were performed; the average values were calculated from five replicates of each experiment.

### RNA extraction and quantitative real-time RT-PCR

Total RNA was extracted from the cultured human liver cancer cells, HUVECs, or frozen liver tissues using the RNeasy mini kit (QIAGEN, Tokyo, Japan) as per the manufacturer’s instructions. Total RNA from each sample was reverse transcribed into cDNA using the high-capacity RNA-to-cDNA kit (Applied Biosystems Inc., Foster City, Calif., USA) as per the manufacturer’s instructions. The mRNA expression was assessed with quantitative real-time PCR using the StepOnePlus real-time PCR system (Applied Biosystems, Foster City, USA) using Fast SYBR Green master mix (Applied Biosystems). Relative gene expression was measured with glyceraldehyde-3-phosphate dehydrogenase (GAPDH) as an internal control. The relative amount of target mRNA in each cycle was evaluated by applying a threshold cycle to the standard curve. The mRNA levels were indicated as a ratio to the negative control. All primers were purchased from Sigma Aldorich (Additional file 1: Table S1).

### Protein extraction and western blotting

Whole cell lysates were prepared from 10^6^ cultured human liver cancer cells using T-PER Tissue Protein Extraction Reagent supplemented with proteinase and phosphatase inhibitors (all Thermo Scientific, Rockford, IL, USA). Fifty micrograms of whole cell lysates were separated by SDS-PAGE; thereafter, they were transferred to a PVDF membrane that was subsequently blocked with 5% bovine serum albumin in Tris-buffered saline + Tween–20 for 1 h. Thereafter, each membrane was incubated overnight with an antibody against p44/42 mitogen-activated protein kinase (MAPK) [extracellular signal-regulated kinase (ERK) 1/2], Phospho-p44/42 MAPK (Erk1/2) (Thr202/Tyr204), Caspase-3, Cleaved caspase-3, and β-Actin (Cell Signaling Technology); washed; and incubated with Amersham ECL IgG and HRP-linked F(ab)2 fragment (GE Healthcare Life Sciences, Piscataway, NJ, USA; 1:5000 dilution). Finally, each membrane was developed using Clarity Western ECL Substrate (BIORAD, Hercules, CA, USA).

### In vitro endothelial tubular formation

In vitro angiogenesis was defined as the formation of capillary-like structures in HUVECs co-cultures with human diploid fibroblasts, as previously described [9]. In brief, 24-well tissue culture plates were coated with Matrigel (Collaborative Biochemical Products, Bedford, MA, USA; 150 mL/well) and allowed to set at 37 °C for 30 min. Thereafter, 2.5 × 10^4^ HUVECs were added to each well and incubated at 37 °C for 20 h under 5% CO_2_ atmosphere and different conditions. Semi-quantitative analysis of tubule formation was performed using NIH ImageJ software (http://imagej.nih.gov/ij/).

### Statistical analyses

The data were subjected to Student’s *t*-test or one-way analysis of variance followed by Bonferroni’s multiple-comparison test, as appropriate. Bartlett’s test was used for determining the homology of variance. Statistical analyses were performed using Prism, version 6.04 (GraphPad Software, La Jolla, CA, USA). All tests were two-tailed, and *P*-values < 0.05 were considered statistically significant.

## Results

### Physical and serological findings in OLETF and LETO rats

All the rats were survived until the end of experiments. Physical and serological data for all the experimental groups of OLETF and LETO rats are presented in Table 1.

**Table 1:** Characteristic features of the experimental rats

Rat	LETO	OLETF			
Treatment	Vehicle	Vehicle	ACR	ARB	ACR + ARB
Body weight (g)	410.0 ± 21.6	498.0 ± 27.3^a^	491.7 ± 33.4^a^	493.8 ± 32.9^a^	480.0 ± 25.0^a^
Triglyceride (mg/dL)	18.0 ± 4.0	83.8 ± 16.0^a^	74.0 ± 10.8^ab^	60.3 ± 2.0^ab^	59.0 ± 11.1^ab^
Glucose (mg/dL)	174.3 ± 17.1	199.6 ± 26.6^a^	189.8 ± 13.9^a^	184.2 ± 12.6^a^	184.3 ± 19.2^a^
Insulin (μU/ml)	8.5 ± 2.8	33.2 ± 13.1^a^	29.9 ± 22.3^a^	35.4 ± 24.3^a^	33.6 ± 15.0^a^
AST (IU/L)	216.7 ± 10.6	239.6 ± 41.4^a^	232.9 ± 32.0^a^	237.4 ± 50.9^a^	242.8 ± 44.2^a^
ALT (IU/L)	51.5 ± 2.0	73.9 ± 14.4^a^	66.4 ± 8.4^a^	67.1 ± 11.1^a^	74.3 ± 6.9^a^
Albumin (g/mL)	3.85 ± 0.15	4.11 ± 0.09	3.93 ± 0.11	3.87 ± 0.13	4.02 ± 0.1
Total bilirubin (mg/dL)	0.100 ± 0.02	0.103 ± 0.02	0.093 ± 0.017	0.095 ± 0.012	0.079 ± 0.017

Data presented as mean ± standard deviation (SD) values. Each group comprises 10 rats. a: *p* < 0.05 vs. LETO with vehicle group. B: p < 0.05 vs. OLETF with vehicle group

According to the underlying characteristics, OLETF rats exhibited obesity, hypertriglyceridemia, hyperglycemia, and hyperinsulinemia along with mild elevation of aspartate and alanine aminotransferase (AST and ALT) as compared to the LETO rats. In the OLETF rats, HOMA-IR was notably higher. Correspondingly, QUICKI, a surrogate marker of insulin sensitivity, was lower than that in the LETO rats (Fig. 1b). Meanwhile, there was no significant difference between the two groups in terms of the serum albumin and total bilirubin levels. Among the OLETF groups, treatment of ACR and/or ARB reduced hypertriglyceridemia; however, the body weight, liver function, and glycemic status remained unchanged (Table 1 and Fig. 1b).

### ACR and ARB ameliorate IR-enhanced growth of GST-P positive pre-neoplastic lesions in diabetic OLETF rats

Initially, in order to determine whether IR stimulates the augmentation of hepatocarcinogenesis, we conducted a histological comparison of the number and size of the DEN-induced GST-P positive pre-neoplastic lesions (GST-P^+^ PNL) between the LETO and OLETF rats (Fig. 1c). Semi-quantitative analyses revealed that both the number and size of hepatic GST-P^+^ PNL in the OLETF rats were higher than those in the LETO rats, indicating that IR promotes hepatocarcinogenesis (Fig. 1d). Then, we evaluated the effects of ACR and/or ARB on the IR-induced exacerbation of hepatocarcinogenesis. In non-diabetic LETO rats, either ACR or ARB significantly decreased the number and size of hepatic GST-P^+^ PNL in accordance with the previous reports (Additional file 2: Figure S1a, b). Interestingly, these suppressive effects were observed in diabetic OLETF rats with greater magnitude than those in LETO rats (Fig. 1c, d). Moreover, the individual administration of ACR or ARB demonstrated weaker effects than the combined administration of ACR and ARB (Fig. 1c, d).

### Effects of ACR and ARB on proliferation, cell cycle, and apoptosis of the DEN-treated liver of OLETF rats and human liver cancer cells

**Fig. 2:**
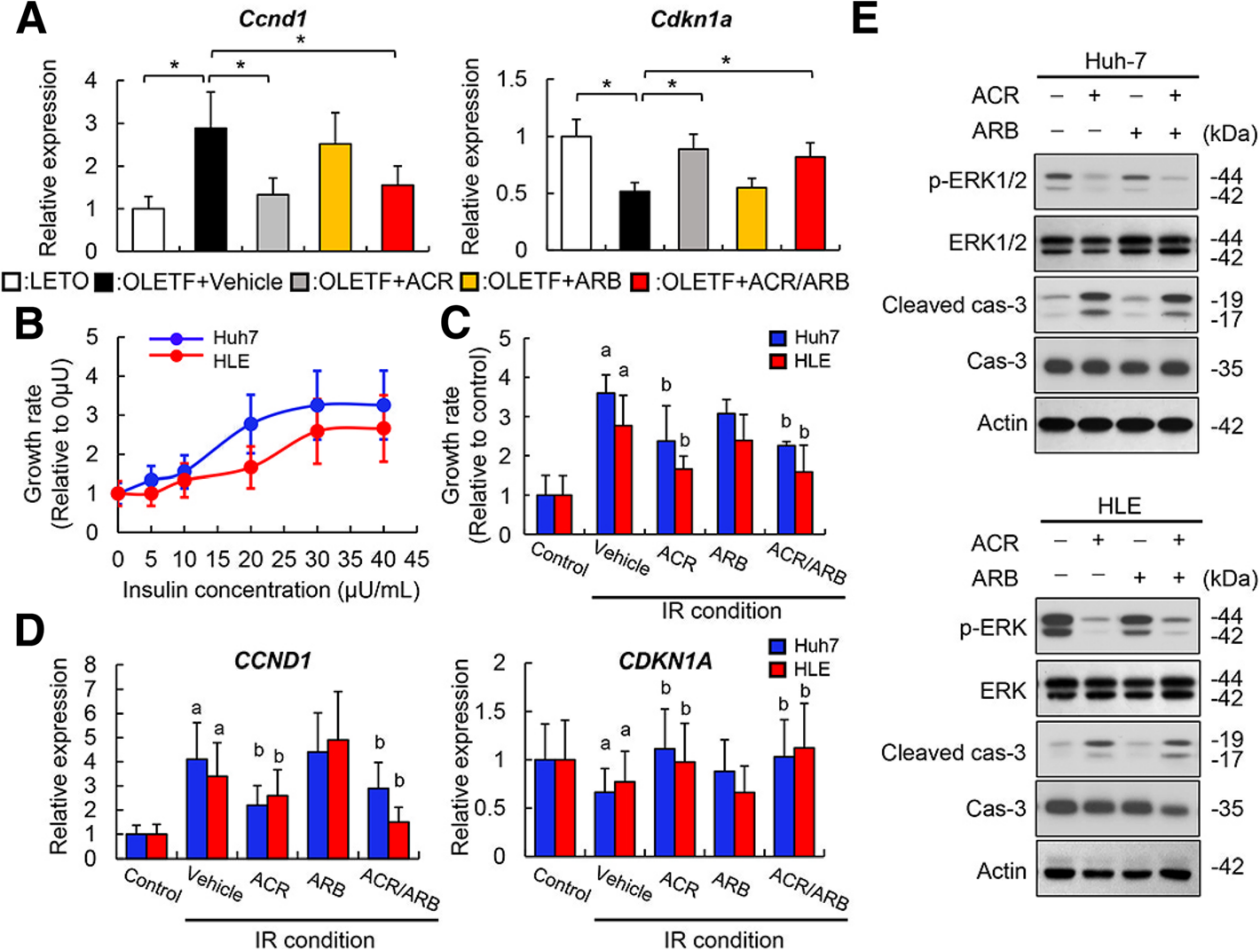
Effects of acyclic retinoid (ACR) and angiotensin-II receptor blocker (ARB) on liver cancer cell growth. **a** Relative mRNA expression levels of rat *Ccnd1* (Left panel) and *Cdkn1a* (Right panel) in the liver of experimental rats. **b** Recombinant human insulin (rh-insulin)-stimulated cell proliferation in human liver cancer cell lines (Huh-7 and HLE) at different concentration. Cell proliferation is indicated as ratio to the data of 0μU. **c** The effects of ACR and/or ARB on the proliferation of human liver cancer lines cultured under the insulin resistance(IR)-mimetic condition. The cells as control were cultured under normal condition. **d** The effects of ACR and/or ARB on the relative mRNA expression levels of human *CCND1* (Left panel) and *CDKN1A* (Right panel) in human liver cancer lines cultured under the IR-mimetic condition. **e** The effects of ACR and/or ARB on ERK phosphorylation and Caspase-3 (Cas-3) cleavage in Huh-7 (Upper panel) and HLE (Lower panel) cultured under the IR-mimetic condition. LETO; Long-Evans Tokushima Otsuka rat, OLETF; Otsuka Long-Evans Tokushima Fatty rat. Relative mRNA expression levels were measured by quantitative RT–PCR (qRT–PCR), and *GAPDH* was used as internal control for qRT–PCR (**a**, **d**). The protein was determined by western blotting, and actin was used as the loading control (**e**). The IR-mimetic condition is defined as the higher concentration of D-(+)-glucose (199.6 mg/dl) and rh-insulin (33.2 μU/ml) (**c**-**e**). ACR (10 μM) and/or ARB (10 μM) were added to each group for in vitro experiments (**c**-**e**). Data are mean ± SD (*n* = 10). *, *P* ≤ 0.01 between each group (**a**); ^a^, *P* ≤ 0.01 as compared with the control group; ^b^, *P* ≤ 0.01 as compared with vehicle group (**c** and **d**)

Several reports have shown that ACR can inhibit HCC cell growth by increasing the cellular levels of p21^CIP1^ and concomitantly decreasing the cyclin D1 levels [24]. Thus, we evaluated the mRNA expressions of these molecules in the liver of DEN-treated rats during tumorigenesis. Quantitative real-time RT-PCR analysis indicated that OLETF rats showed decreased *Cdkn1a* and increased *Ccnd1* mRNA levels compared to LETO rats; further, these altered expressions were inhibited by ACR treatment (Fig. 2a). In contrast, ARB treatment had no influence on these mRNA levels. Subsequently, we assessed the effects of ACR and ARB on in vitro cell growth in human liver cancer cells under both normal and IR condition. First, we examined the effects of both agents on the proliferation of two human liver cancer lines, Huh7 and HLE, under the normal culture condition. ACR inhibited cell proliferation of both of two lines, while ARB did not (Additional file 3: Figure S2a). Next, to evaluate the effect on cell growth under IR condition, we examined the inducible activity of rh-insulin on cell proliferation for optimizing the IR-mimetric condition. As shown in Fig. 2b, rh-insulin induced cell proliferation of two human liver cancer lines in a dose-dependent manner. Thereafter, we generated IR-mimetic conditional media by modulating higher concentrations of D-(+)-glucose (199.6 mg/dl) and rh-insulin (33.2 μU/ml), almost corresponding to the serum level in the OLETF rats for evaluating the effects of ACR and ARB on cell proliferation under the IR condition. It is noteworthy that ACR treatment considerably inhibited IR-stimulated cell proliferation of both the liver cancer lines (Fig. 2c). Consistent with the in vivo results, IR condition stimulated the downregulation of *CDKN1A* and upregulation of *CCDN1*. These alteration in mRNA levels were inhibited by ACR treatment (Fig. 2d). Moreover, ACR suppressed ERK1/2 phosphorylation and facilitated caspase-3 cleavage in both human liver cancer lines (Fig. 2e). These in vitro findings support that ACR directly inhibits cell proliferation by inducing cell cycle arrest and apoptosis in liver cancer cells under the IR condition. There was no difference in the degree of inhibitory effect of ACR on liver cancer cell growth between normal culture condition and IR condition (Fig. 2c and Additional file 3: Figure S2a). As in the DEN-treated liver tissue, ARB treatment did not directly influence cell growth or survival in liver cancer lines even under the IR condition.

### Effects of ACR and ARB on intrahepatic angiogenesis in DEN-treated liver of OLETF rats and vascular endothelial in vitro growth

**Fig. 3:**
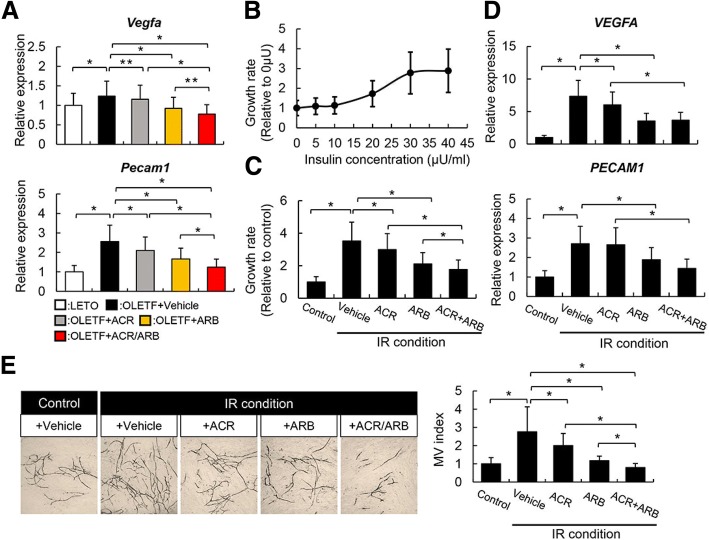
Effects of acyclic retinoid (ACR) and angiotensin-II receptor blocker (ARB) on vascular endothelial growth. **a** Relative mRNA expression levels of rat *Vegfa* (Upper panel) and *Pecam1* (Lower panel) in the liver of experimental rats. **b** Recombinant human insulin (rh-insulin)-stimulated cell proliferation in human umbilical vascular endothelial cell (HUVEC) at different concentration. Cell proliferation is indicated as ratio to the data of 0μU. **c** The effects of ACR and/or ARB on the proliferation of HUVEC cultured under the insulin resistance(IR)-mimetic condition. The cells as control were cultured under normal condition. **d** The effects of ACR and/or ARB on the relative mRNA expression levels of human *VEGFA* (Upper panel) and *PECAM1* (Lower panel) in HUVEC cultured under the IR-mimetic condition. **e** The effects of ACR and/or ARB on in vitro endothelial tubular formation (Left panel; representative pictures, Right panel; Semi-quantitative analysis). LETO; Long-Evans Tokushima Otsuka rat, OLETF; Otsuka Long-Evans Tokushima Fatty rat. Relative mRNA expression levels were measured by quantitative RT–PCR (qRT–PCR), and *GAPDH* was used as internal control for qRT–PCR (**a**, **d**). The IR-mimetic condition is defined as the higher concentration of D-(+)-glucose (199.6 mg/dl) and rh-insulin (33.2 μU/ml) (**c**-**e**). ACR (10 μM) and/or ARB (10 μM) were added to each group for in vitro experiments (**c**-**e**). Data are mean ± SD (*n* = 10). *, *P* ≤ 0.01; **, *P* ≤ 0.05 between each group

Considerable evidence suggests that neovascularization plays a crucial role in hepatocarcinogenesis, especially in the presence of IR; therefore, we then investigated angiogenesis in the liver tissue of DEN-treated rats [9]. Quantitative real-time RT-PCR analysis revealed that mRNA levels of *Vegfa* and *Pecam1*, representative molecules associated with angiogenesis, were raised in the liver of OLETF rats (Fig. 3a). ACR or ARB treatment suppressed the upregulation of *Vegfa* and *Pecam1* mRNA levels, while the combination treatment exerted a much stronger suppressive effect than each monotherapy (Fig. 3a). We further evaluated the in vitro cell growth of the vascular endothelial cell lines, HUVEC, to validate the effects of both the agents on the angiogenic activity. Similar to the experiment for liver cancer cells, we first assessed the effects of ACR and ARB on the proliferation of HUVEC cultured under normal condition. As shown in Additional file 3: Figure S2b, both ACR and ARB suppressed HUVEC proliferation. We next confirmed that rh-insulin could induce HUVEC proliferation in a dose-dependent manner (Fig. 3b). IR-mimetic condition markedly promoted the proliferation of HUVECs and liver cancer cells. Further, both ACR and ARB inhibited IR-induced HUVEC proliferation with single treatment (Fig. 3c). Comparing the magnitude of these antiproliferative effects of ACR and ARB, there was no difference between normal culture condition and IR condition. It is noteworthy that the combination treatment exerted a more potent antiproliferative effect on HUVECs (Fig. 3c). These effects coincided with the downregulation of *VEGFA* and *PECAM1* mRNA levels in HUVEC cultured under the IR-mimic condition (Fig. 3d). Moreover, we examined whether both agents affect endothelial tubular formation. As shown in Fig. 3e, ARB strongly inhibited tubular formation, and ACR significantly suppressed EC tubular formation; further, combined treatment with ACR and ARB showed additive inhibitory effect on tubular formation compared with each single treatment. Thus, an anticancer effect of ACR and ARB is at least partially involved in their antiangiogenic activities.

### Effects of ACR and ARB on oxidative DNA damage and lipid peroxidation in the liver of OLETF rats

**Fig. 4:**
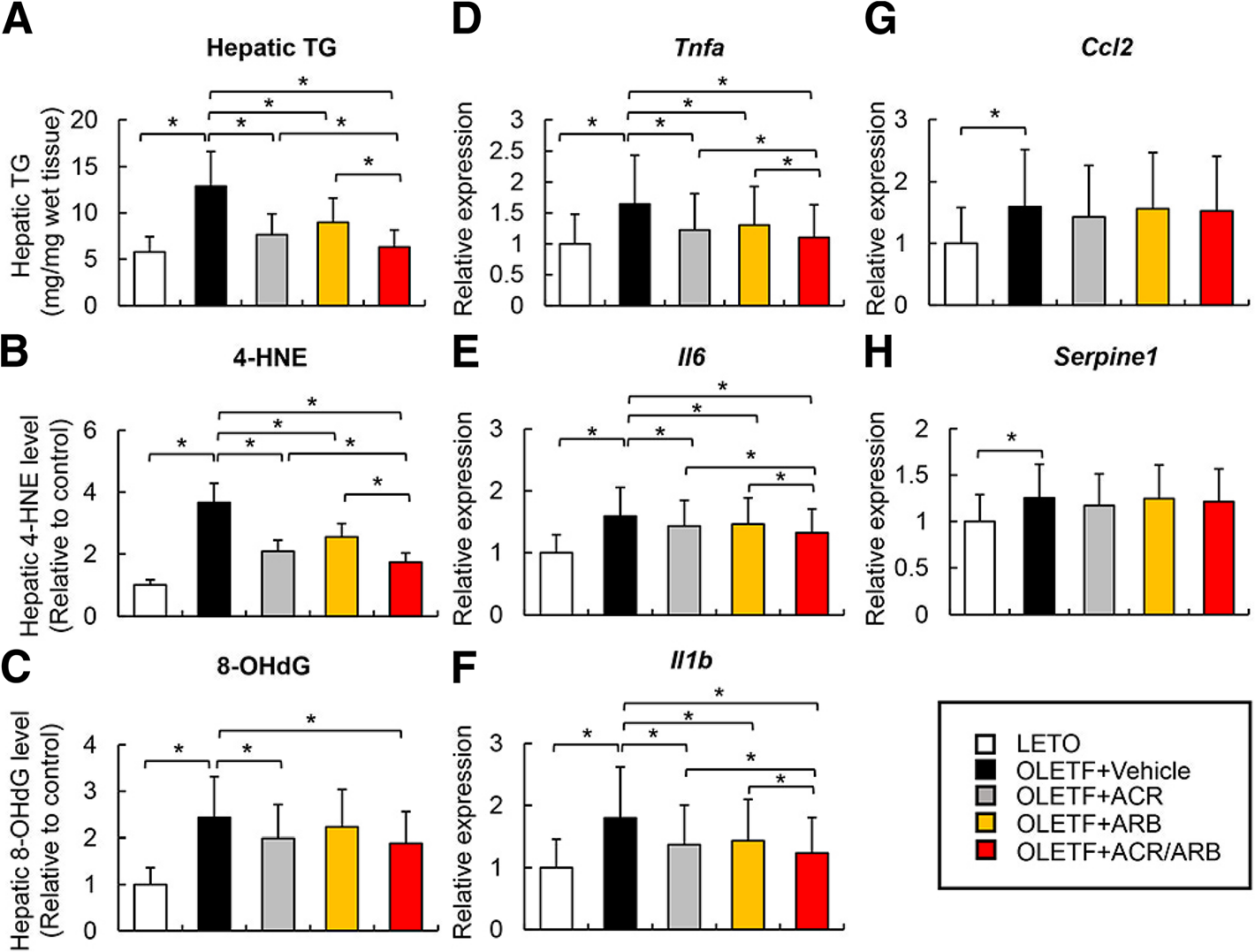
Effects of acyclic retinoid (ACR) and angiotensin-II receptor blocker (ARB) on liver steatosis, oxidative stress and inflammation. **a**-**c** Hepatic levels of triglyceride (TG) (**a**), 4-Hydroxynonenal (4-HNE) (**b**) and 8-hydroxy-2′-deoxyguanosine (8-OHdG) (**c**) in experimental rats. **d**-**h** Relative mRNA expression levels of rat *Tnfa* (**d**), *Il6* (**e**), *Il1b* (**f**), *Ccl2* (**g**) and *Serpine1* (**h**) in the liver of experimental rats. LETO; Long-Evans Tokushima Otsuka rat, OLETF; Otsuka Long-Evans Tokushima Fatty rat. 4-HNE and 8-OHdG levels are indicated as ratio to LETO control group (**b** and **c**). Relative mRNA expression levels were measured by quantitative RT–PCR (qRT–PCR), and *GAPDH* was used as internal control for qRT–PCR (**d**-**h**). Data are mean ± SD (*n* = 10). *, *P* ≤ 0.01 between each group

As reactive oxygen species are critical for the development of hepatocarcinogenesis, we assessed the effect of ACR and ARB on lipid peroxidation and oxidative DNA damage. First, we measured the hepatic content of triglycerides in the DEN-treated rats. The hepatic triglyceride level was higher in the OLETF rats compared to that in the LETO rats and was lowered following ACR treatment (Fig. 4a). Similarly, ARB treatment also decreased the triglyceride levels in the liver of the OLETF rats, while combined treatment showed greater reduction than each single treatment (Fig. 4a). Corresponding to the decrease in hepatic triglycerides, the levels of hepatic 4-HNE, a lipid peroxidation marker, were lowered in ACR- and/or ARB- treated OLETF rats (Fig. 4b). Thereafter, we evaluated the level of hepatic 8-OHdG, an indicator of oxidative DNA damage. As shown in Fig. 4c, hepatic 8-OHdG level was lower in ACR-treated OLETF rats than in the vehicle-treated rats, while there was no significant change in the ARB-treated rats. In addition to oxidative stress, an inflammatory state based on IR and lipid accumulation also plays a crucial role in obesity-associated hepatocarcinogenesis. Thus, we further examined the effects of ACR and ARB on the levels of the proinflammatory cytokines, *Tnfa*, *Il6*, *Il1b*, *Ccl2,* and *Serpine1,* as well as the mRNA levels in the liver of DEN-treated OLETF rats. Both treatments (ACR and ARB) lowered the hepatic mRNA levels of *Tnfa*, *Il6,* and *Il1b* (Fig. 4d, f). The hepatic expressions of these proinflammatory cytokines were lower following combined treatment than after each single treatment (Fig. 4d, f). Nevertheless, neither agent altered the hepatic mRNA levels of *Ccl2* and *Serpine1* (Fig. 4g, h).

## Discussion

Currently, the increasing prevalence of obesity is recognized as one of the most serious global public health issues. Among all obesity-related medical abnormalities, IR is particularly closely associated with the progression of various types of malignancies, including breast, colorectal, and pancreatic cancers as well as HCC [4–8, 25–27]. Numerous evidences show that IR causatively plays a key role in HCC development. Although the insulin signaling pathway is known to be responsible for tumor initiation, it insufficiently contributes to hepatocarcinogenesis by itself. Actually, hepatocytes may acquire the capacity to adapt insulin-dependent mechanisms for their proliferation and survival during chronic liver injury, and sequentially may promote premalignant transformation and tumor growth [28]. Moreover, the IR status fosters a hepatic microenvironment that favors the propagation of premalignant and malignant cells, such as angiogenesis, oxidative stress, and chronic inflammation [29–32]. Our studies on mice models show that the IR status may facilitate hepatocarcinogenesis by stimulating hepatic neovascularization and VEGF expression; both high glucose and insulin could prime in vitro endothelial tubular formation via ERK1/2 phosphorylation [9]. IR also plays a crucial role in the production of oxidative stress in the liver, thus upregulating the proinflammatory cytokines [31–34]. These cytokines stimulate chronic liver inflammation with hepatic steatosis, and consequently lead to liver tumorigenesis. IR-stimulated oxidative stress also induces DNA adduct formation to initiate tumor formation. These mechanistic evidences suggest that IR and its related metabolic alterations could be crucial chemopreventive targets for obesity-related hepatocarcinogenesis.

The present study distinctly demonstrated that clinically equivalent doses of ACR or ARB successfully ameliorated DEN-induced hepatocarcinogenesis in diabetic OLETF rats. Recent researches have progressively demonstrated the mechanistic insights in the regulatory effects of ACR on RXRα. Therefore, ACR could activate RXRα not only directly by acting as its ligand, but also indirectly by inhibiting the Ras-ERK pathway that devitalizes RXRα by phosphorylating it [35]. Thus, current in vitro results also revealed that ACR treatment inhibited human HCC cells growth with ERK1/2 dephosphorylation. In contrast, losartan, used as ARB, did not affect HCC cell growth in this study. It is noteworthy that telmisartan, another kind of ARB, reportedly exerts antiproliferative effect on human HCC cell lines by inducing cell cycle arrest [36]. A major underlying reason for this discrepancy could be the difference in the pharmacological activity of losartan and telmisartan. For example, telmisartan is known to possess peroxisome-proliferator-activated-receptor (PPAR) γ-activating action; however, losartan does not possess it [37]. PPARγ activation contributes, at least partially, to HCC cell growth inhibition; therefore, this additive action of telmisartan may be involved in HCC growth suppression.

It is noteworthy that the despite ineffectiveness of ARB on HCC cells, combined ACR and ARB treatment showed a marked synergistic inhibitory effect on the development of pre-neoplastic lesions in OLETF livers. Thus, to elucidate the underlying mechanism of this synergistic effect, we focused on the antiangiogenic properties of ACR and ARB. Different angiostatic agents, including ARB, are reported to frequently exert additive or synergistic antitumor effects in combination with each other [38]. Therapies that target tumor vessels are proven beneficial for cancer treatment in experimental models [39]. However, some studies suggest that treatment with a single antiangiogenic agent may be insufficient for complete suppression of tumor angiogenesis because the compensatory activation of proangiogenic factors was mostly found following treatment with a single angiostatic agent [38, 40]. Actually, our previous studies has demonstrated that monotherapy with an ACE-I or vitamin K2 could exert anticancer effect in rat hepatocarcinogenesis models, although either treatment alone did not prevent HCC recurrence in clinical practice. Remarkably, the combination treatment of both the agents exclusively achieved efficient prevention of cumulative HCC recurrence after curative therapy, along with VEGF suppression [41]. In the current study, ARB treatment expectedly exerted antiangiogenic activities in the liver of OLETF rats, as in our previous reports. However, it was surprising to observe suppressed intrahepatic angiogenesis in ACR-treated rats. Our in vitro study also confirmed that ACR and ARB attenuated IR-induced vascular endothelial cell proliferation and tubular formation. A recent report demonstrated that bexarotene, a ligand of RXRα, significantly inhibited HUVEC morphogenesis, proliferation, and migration [42]. Moreover, LGD1069 (Targretin®), a selective RXR ligand, also exerted antiangiogenic effects by suppressing the expression of Runx2, indicating that ACR attenuates vascular endothelial growth mainly by acting as an RXRα ligand in our model [43].

Intriguingly, the results from the present study show that ACR and ARB were likely to improve in vivo hepatocarcinogenesis more potently than in vitro cancer cell and HUVEC proliferation. Following such discrepant findings, we assessed the effects on hepatic lipid metabolism and oxidative stress to identify additional effects of both agents. ACR is reported to efficiently protect from the acceleration of lipogenesis independently of glucose metabolism in DEN-treated mice liver [44]. In addition, several basic studies have demonstrated that ARB reduced hepatic steatosis [23, 45, 46]. According to these evidences, our data showed that ACR and ARB reduced hepatic triglyceride accumulation with reduced serum hypertriglyceridemia; however, it did not affect the glycemic status of OLETF rats. ACR and ARB also suppressed hepatic lipid peroxidation coincidently while inhibiting lipogenesis; however, only ACR reduced the oxidative DNA damage in the liver of OLETF rats. Further investigation is warranted for a deeper understanding of the functional mechanism underlying the differential antioxidant effects of ACR and ARB. IR and hepatic lipid accumulation also stimulate inflammatory changes in the liver. ACR and ARB reduced the hepatic expression levels of *Tnfa*, *Il6*, and *Il1b* mRNA in OLETF rats with improved hepatic steatosis. Tumor necrosis factor (TNF)-α is a key mediator of IR-induced interleukin (IL)-6 production and hepatocarcinogenesis, and IL-6 plays a crucial role in HCC development via STAT3 activation [47]. Aberrant activation of Janus kinase (JAK)-STAT signaling pathways is critical for HCC development; therefore, the anti-inflammatory properties of ACR and ARB partially contribute toward protection from hepatocarcinogenesis in DEN-treated OLETF rats by blocking STAT3 activation. On the other hand, neither ACR nor ARB affected oxidative DNA damage, lipid peroxidation and inflammatory cytokine levels in LETO rats model. (data not shown). Thus, we speculate that ACR and ARB could potentiate their anticancer effects with improvement these microenvironmental status.

ACR and ARB have been demonstrated to exert multiple pharmacological activities for efficiently preventing hepatocarcinogenesis. Similar to ARB, ACR also commonly exerts synergistic effects in combination with other clinically available drugs, including interferon-β, valproic acid, vitamin K2, trastuzumab, and bevacizumab [48–51]. In addition to these agents, recent xenograft analysis has demonstrated that branched-chain amino acids (BCAA) may also be used as a candidate partner for peretinoin-based cocktail therapy against HCC [35]. Therefore, the combination of both agents is likely to be one of the best therapies for chemoprevention against HCC. However, we need to ponder over the effect of ACR and ARB on IR, because the IR status, as estimated using HOMA-IR and QUICKI, were not changed by treatment with ACR nor ARB in this study.

Nevertheless, further study with long-term administration of both agents may prove that the IR-modulating effect of this combination regimen plays a key role in the chemoprevention of IR-based hepatocarcinogenesis.

## Conclusions

In sum, we elucidated that ACR and ARB exerted a combined inhibitory effect on hepatocarcinogenesis along with the inhibition of angiogenesis, oxidative stress, and chronic inflammation. It is noteworthy that these inhibitory effects were achieved at clinically relevant low doses without causing any serious adverse events. Therefore, this combination regimen may eventually emerge as a potential new therapeutic strategy for chemoprevention against IR-based hepatocarcinogenesis.

## Additional files

Additional file 1: **Table S1.** List of primers for quantitative RT-PCR. All reagents were purchased from Sigma Aldorich and RefSeq ID are provided (DOCX 15 kb)

Additional file 2: **Figure S1.** Effects of acyclic retinoid (ACR) and angiotensin-II receptor blocker (ARB) on hepatocarcinogenesis in non-diabetic rats. (A) Representative microphotographs of glutathione S-transferase (GST-P)-positive preneoplastic foci. (B) Number of GST-P positive neoplastic lesions (GST-P^+^ PNL) per square centimeter (Upper panel) and relative size of GST-P^+^ PNL (Lower panel). LETO; Long-Evans Tokushima Otsuka rat, Data are mean ± SD (*n* = 10). **, *P* ≤ 0.05 between each group (B). (PDF 927 kb)

Additional file 3: **Figure S2.** Effects of acyclic retinoid (ACR) and angiotensin-II receptor blocker (ARB) on liver cancer cell and human umbilical vein endothelial cell (HUVEC) growth cultured under normal condition.(A) The effects of ACR and/or ARB on the proliferation of human liver cancer lines cultured under condition. (B) The effects of ACR and/or ARB on the proliferation of HUVECs cultured under condition. Data are mean ± SD (*n* = 10). ^a^, *P* ≤ 0.01 as compared with the vehicle-mediated group (A); **, *P* ≤ 0.05 between each group (B). (PDF 211 kb)

## Abbreviations

4-HNE: 4-Hydroxynonenal
8-OHdG: 8-hydroxy-2′-deoxyguanosine
ACE-I: Angiotensin-converting enzyme inhibitor
ACR: Acyclic retinoid
ALT: Alanine aminotransferase
ARB: Angiotensin-II type1 receptor blocker
AST: Aspartate aminotransferase
BCAA: Branched-chain amino acids
DEN: Diethylnitrosamine
ERK: Extracellular signal-regulated kinase
GAPDH: Glyceraldehyde-3-phosphate dehydrogenase
GST-P: Placental glutathione S-transferase
GST-P^+^ PNL: GST-P positive pre-neoplastic lesions
HCC: Hepatocellular carcinoma
HOMA-IR: Homeostasis model assessment-insulin resistance
HUVEC: Human umbilical vascular endothelial cell
IL: Interleukin
IR: Insulin resistance
JAK: Janus kinase
LETO: Long-Evans Tokushima Otsuka
MAPK: Mitogen-activated protein kinase
NAFLD: Nonalcoholic fatty liver disease
NASH: Nonalcoholic steatohepatitis
OLETF: Otsuka Long-Evans Tokushima Fatty
PPAR: Peroxisome-proliferator-activated-receptor
QUICKI: Quantitative insulin sensitivity check index
RAAS: Renin-angiotensin-aldosterone system
RAR: Retinoic acid receptor
rh-insulin: Recombinant human insulin
RXR: Retinoid X receptor
STAT: Signal transducers and activators of transcription
T2DM: Type2 diabetes mellitus
TNF: Tumor necrosis factor
VEGF: Vascular endothelial growth factor

## References

[1] Torre LA, Bray F, Siegel RL, Ferlay J, Lortet-Tieulent J, Jemal A (2015). Global cancer statistics, 2012. CA Cancer J Clin.

[2] Castells A, Bruix J, Bru C, Fuster J, Vilana R, Navasa M (1993). Treatment of small hepatocellular carcinoma in cirrhotic patients: a cohort study comparing surgical resection and percutaneous ethanol injection. Hepatology.

[3] Llovet JM, Schwartz M, Mazzaferro V (2005). Resection and liver transplantation for hepatocellular carcinoma. Semin Liver Dis.

[4] Siddique A, Kowdley KV (2011). Insulin resistance and other metabolic risk factors in the pathogenesis of hepatocellular carcinoma. Clinics in liver disease.

[5] Loftfield E, Freedman ND, Lai GY, Weinstein SJ, McGlynn KA, Taylor PR (2016). Higher glucose and insulin levels are associated with risk of liver Cancer and chronic liver disease mortality among men without a history of diabetes. Cancer Prev Res.

[6] Simon Tracey G., King Lindsay Y., Chong Dawn Q., Nguyen Long H., Ma Yanan, VoPham Trang, Giovannucci Edward L., Fuchs Charles S., Meyerhardt Jeffrey A., Corey Kathleen E., Khalili Hamed, Chung Raymond T., Zhang Xuehong, Chan Andrew T. (2018). Diabetes, metabolic comorbidities, and risk of hepatocellular carcinoma: Results from two prospective cohort studies. Hepatology.

[7] El-Serag HB, Tran T, Everhart JE (2004). Diabetes increases the risk of chronic liver disease and hepatocellular carcinoma. Gastroenterology.

[8] Davila JA, Morgan RO, Shaib Y, McGlynn KA, El-Serag HB (2005). Diabetes increases the risk of hepatocellular carcinoma in the United States: a population based case control study. Gut.

[9] Kaji K, Yoshiji H, Kitade M, Ikenaka Y, Noguchi R, Yoshii J (2008). Impact of insulin resistance on the progression of chronic liver diseases. Int J Mol Med.

[10] Tateishi R, Okanoue T, Fujiwara N, Okita K, Kiyosawa K, Omata M (2015). Clinical characteristics, treatment, and prognosis of non-B, non-C hepatocellular carcinoma: a large retrospective multicenter cohort study. J Gastroenterol.

[11] Younossi ZM, Otgonsuren M, Henry L, Venkatesan C, Mishra A, Erario M (2015). Association of nonalcoholic fatty liver disease (NAFLD) with hepatocellular carcinoma (HCC) in the United States from 2004 to 2009. Hepatology.

[12] Araki H, Shidoji Y, Yamada Y, Moriwaki H, Muto Y (1995). Retinoid agonist activities of synthetic geranyl geranoic acid derivatives. Biochem Biophys Res Commun.

[13] Muto Y, Moriwaki H, Saito A (1999). Prevention of second primary tumors by an acyclic retinoid in patients with hepatocellular carcinoma. N Engl J Med.

[14] Okita K, Izumi N, Matsui O, Tanaka K, Kaneko S, Moriwaki H (2015). Peretinoin after curative therapy of hepatitis C-related hepatocellular carcinoma: a randomized double-blind placebo-controlled study. J Gastroenterol.

[15] Kagawa M, Sano T, Ishibashi N, Hashimoto M, Okuno M, Moriwaki H (2004). An acyclic retinoid, NIK-333, inhibits N-diethylnitrosamine-induced rat hepatocarcinogenesis through suppression of TGF-alpha expression and cell proliferation. Carcinogenesis.

[16] Shimizu M, Shirakami Y, Imai K, Takai K, Moriwaki H (2012). Acyclic retinoid in chemoprevention of hepatocellular carcinoma: targeting phosphorylated retinoid X receptor-alpha for prevention of liver carcinogenesis. J Carcinog.

[17] Funaki M, Kitabayashi J, Shimakami T, Nagata N, Sakai Y, Takegoshi K (2017). Peretinoin, an acyclic retinoid, inhibits hepatocarcinogenesis by suppressing sphingosine kinase 1 expression in vitro and in vivo. Sci Rep.

[18] Shimizu M, Sakai H, Shirakami Y, Iwasa J, Yasuda Y, Kubota M (2011). Acyclic retinoid inhibits diethylnitrosamine-induced liver tumorigenesis in obese and diabetic C57BLKS/J- +(db)/+Lepr(db) mice. Cancer Prev Res.

[19] Yoshiji H, Yoshii J, Ikenaka Y, Noguchi R, Tsujinoue H, Nakatani T (2002). Inhibition of renin-angiotensin system attenuates liver enzyme-altered preneoplastic lesions and fibrosis development in rats. J Hepatol.

[20] Yoshiji H, Yoshii J, Ikenaka Y, Noguchi R, Yanase K, Tsujinoue H (2002). Suppression of the renin-angiotensin system attenuates vascular endothelial growth factor-mediated tumor development and angiogenesis in murine hepatocellular carcinoma cells. Int J Oncol.

[21] Yang Y, Wei RB, Xing Y, Tang L, Zheng XY, Wang ZC (2013). A meta-analysis of the effect of angiotensin receptor blockers and calcium channel blockers on blood pressure, glycemia and the HOMA-IR index in non-diabetic patients. Metab Clin Exp.

[22] Yoshiji H, Noguchi R, Kaji K, Ikenaka Y, Shirai Y, Namisaki T (2010). Attenuation of insulin-resistance-based hepatocarcinogenesis and angiogenesis by combined treatment with branched-chain amino acids and angiotensin-converting enzyme inhibitor in obese diabetic rats. J Gastroenterol.

[23] Kato J, Koda M, Kishina M, Tokunaga S, Matono T, Sugihara T (2012). Therapeutic effects of angiotensin II type 1 receptor blocker, irbesartan, on non-alcoholic steatohepatitis using FLS-Ob/Ob male mice. Int J Mol Med.

[24] Suzui M, Masuda M, Lim JT, Albanese C, Pestell RG, Weinstein IB (2002). Growth inhibition of human hepatoma cells by acyclic retinoid is associated with induction of p21(CIP1) and inhibition of expression of cyclin D1. Cancer Res.

[25] Peairs KS, Barone BB, Snyder CF, Yeh HC, Stein KB, Derr RL (2011). Diabetes mellitus and breast cancer outcomes: a systematic review and meta-analysis. J Clin Oncol : official journal of the American Society of Clinical Oncology.

[26] Colangelo LA, Gapstur SM, Gann PH, Dyer AR, Liu K (2002). Colorectal cancer mortality and factors related to the insulin resistance syndrome. Cancer Epidemiol, Biomarkers Prev : a publication of the American Association for Cancer Research, cosponsored by the American Society of Preventive Oncology.

[27] Carreras-Torres R, Johansson M, Gaborieau V, Haycock PC, Wade KH, Relton CL, et al. The role of obesity, type 2 diabetes, and metabolic factors in pancreatic Cancer: a Mendelian randomization study. J Natl Cancer Inst. 2017;109(9). 10.1093/jnci/djx012. 10.1093/jnci/djx012 PMC5721813 28954281

[28] Chettouh H, Lequoy M, Fartoux L, Vigouroux C, Desbois-Mouthon C (2015). Hyperinsulinaemia and insulin signalling in the pathogenesis and the clinical course of hepatocellular carcinoma. Liver Int: official journal of the International Association for the Study of the Liver.

[29] Escudero CA, Herlitz K, Troncoso F, Guevara K, Acurio J, Aguayo C (2017). Pro-angiogenic role of insulin: from physiology to pathology. Front Physiol.

[30] Kaji K, Yoshiji H, Ikenaka Y, Noguchi R, Aihara Y, Shirai Y (2012). Possible involvement of angiogenesis in chronic liver diseases: interaction among renin-angiotensin-aldosterone system, insulin resistance and oxidative stress. Curr Med Chem.

[31] Esposito K, Nappo F, Marfella R, Giugliano G, Giugliano F, Ciotola M (2002). Inflammatory cytokine concentrations are acutely increased by hyperglycemia in humans: role of oxidative stress. Circulation.

[32] Xu H, Barnes GT, Yang Q, Tan G, Yang D, Chou CJ (2003). Chronic inflammation in fat plays a crucial role in the development of obesity-related insulin resistance. J Clin Invest.

[33] Bertrand F, Philippe C, Antoine PJ, Baud L, Groyer A, Capeau J (1995). Insulin activates nuclear factor kappa B in mammalian cells through a Raf-1-mediated pathway. J Biol Chem.

[34] Manowsky J, Camargo RG, Kipp AP, Henkel J, Puschel GP (2016). Insulin-induced cytokine production in macrophages causes insulin resistance in hepatocytes. Am J Physiol Endocrinol Metab.

[35] Matsushima-Nishiwaki R, Okuno M, Adachi S, Sano T, Akita K, Moriwaki H (2001). Phosphorylation of retinoid X receptor alpha at serine 260 impairs its metabolism and function in human hepatocellular carcinoma. Cancer Res.

[36] Oura K, Tadokoro T, Fujihara S, Morishita A, Chiyo T, Samukawa E (2017). Telmisartan inhibits hepatocellular carcinoma cell proliferation in vitro by inducing cell cycle arrest. Oncol Rep.

[37] Amano Y, Yamaguchi T, Ohno K, Niimi T, Orita M, Sakashita H (2012). Structural basis for telmisartan-mediated partial activation of PPAR gamma. Hypertens Res: official journal of the Japanese Society of Hypertension.

[38] Kerbel RS (2001). Clinical trials of antiangiogenic drugs: opportunities, problems, and assessment of initial results. J Clin Oncol : official journal of the American Society of Clinical Oncology..

[39] Saif MW (2006). Anti-angiogenesis therapy in pancreatic carcinoma. JOP : Journal of the pancreas.

[40] Scappaticci FA, Smith R, Pathak A, Schloss D, Lum B, Cao Y (2001). Combination angiostatin and endostatin gene transfer induces synergistic antiangiogenic activity in vitro and antitumor efficacy in leukemia and solid tumors in mice. Mol Ther: the journal of the American Society of Gene Therapy.

[41] Yoshiji H, Noguchi R, Toyohara M, Ikenaka Y, Kitade M, Kaji K (2009). Combination of vitamin K2 and angiotensin-converting enzyme inhibitor ameliorates cumulative recurrence of hepatocellular carcinoma. J Hepatol.

[42] Escudero P, Navarro A, Ferrando C, Furio E, Gonzalez-Navarro H, Juez M (2015). Combined treatment with bexarotene and rosuvastatin reduces angiotensin-II-induced abdominal aortic aneurysm in apoE(−/−) mice and angiogenesis. Br J Pharmacol.

[43] Fu J, Wang W, Liu YH, Lu H, Luo Y (2011). In vitro anti-angiogenic properties of LGD1069, a selective retinoid X-receptor agonist through down-regulating Runx2 expression on human endothelial cells. BMC Cancer.

[44] Qin XY, Tatsukawa H, Hitomi K, Shirakami Y, Ishibashi N, Shimizu M (2016). Metabolome analyses uncovered a novel inhibitory effect of acyclic retinoid on aberrant lipogenesis in a mouse Diethylnitrosamine-induced hepatic tumorigenesis model. Cancer Prev Res.

[45] Hirose A, Ono M, Saibara T, Nozaki Y, Masuda K, Yoshioka A (2007). Angiotensin II type 1 receptor blocker inhibits fibrosis in rat nonalcoholic steatohepatitis. Hepatology.

[46] Montez P, Vazquez-Medina JP, Rodriguez R, Thorwald MA, Viscarra JA, Lam L (2012). Angiotensin receptor blockade recovers hepatic UCP2 expression and aconitase and SDH activities and ameliorates hepatic oxidative damage in insulin resistant rats. Endocrinology.

[47] Toffanin S, Friedman SL, Llovet JM (2010). Obesity, inflammatory signaling, and hepatocellular carcinoma-an enlarging link. Cancer Cell.

[48] Obora A, Shiratori Y, Okuno M, Adachi S, Takano Y, Matsushima-Nishiwaki R (2002). Synergistic induction of apoptosis by acyclic retinoid and interferon-beta in human hepatocellular carcinoma cells. Hepatology.

[49] Tatebe H, Shimizu M, Shirakami Y, Sakai H, Yasuda Y, Tsurumi H (2009). Acyclic retinoid synergises with valproic acid to inhibit growth in human hepatocellular carcinoma cells. Cancer Lett.

[50] Kanamori T, Shimizu M, Okuno M, Matsushima-Nishiwaki R, Tsurumi H, Kojima S (2007). Synergistic growth inhibition by acyclic retinoid and vitamin K2 in human hepatocellular carcinoma cells. Cancer Sci.

[51] Kubota M, Shimizu M, Baba A, Ohno T, Kochi T, Shirakami Y (2014). Combination of bevacizumab and acyclic retinoid inhibits the growth of hepatocellular carcinoma xenografts. J Nutr Sci Vitaminol.

