# Supplementary material for: Acyclic retinoid and angiotensin-II receptor blocker exert a combined protective effect against diethylnitrosamine-induced hepatocarcinogenesis in diabetic OLETF rats
Source: BMC Cancer. 2018 Nov 26;18:1164. doi: 10.1186/s12885-018-5099-6 (PMC6260898; doi:10.1186/s12885-018-5099-6)
Supplement: Supplementary file 1 — Table S1. List of primers for quantitative RT-PCR. All reagents were purchased from Sigma Aldorich and RefSeq ID are provided (DOCX 15 kb) [file 12885_2018_5099_MOESM1_ESM.docx]

**Supplementary table. 1 List of primers for quantitative RT-PCR, all reagents were purchased from Sigma Aldorich and RefSeq ID are provided**

| **Gene** | **Probe/Primer set** |
| --- | --- |
| **Rat** | |
| *Ccnd1* | NM_171992 |
| *Cdkn1a* | NM_080782 |
| *Vegfa* | NM_ 001110334 |
| *Pecam1* | NM_031591 |
| *Tnfa* | NM_012675 |
| *II6* | NM_012589 |
| *Il1b* | NM_031512 |
| *Ccl2* | NM_031530 |
| *Serpine1* | NM_012620 |
| *Gapdh* | NM_ 001244854 |
| **Human** | |
| *CCND1* | NM_053056 |
| *CDKN1A* | NM_000389 |
| *VEGFA* | NM_ 001204384 |
| *PECAM1* | NM_000442 |
| *GAPDH* | NM_002046 |
