# Supplementary figures and images for: Acyclic retinoid and angiotensin-II receptor blocker exert a combined protective effect against diethylnitrosamine-induced hepatocarcinogenesis in diabetic OLETF rats
Source: BMC Cancer. 2018 Nov 26;18:1164. doi: 10.1186/s12885-018-5099-6 (PMC6260898; doi:10.1186/s12885-018-5099-6)

**A**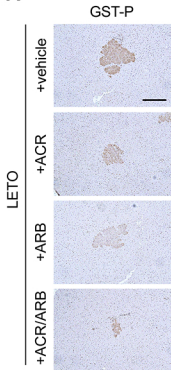**B**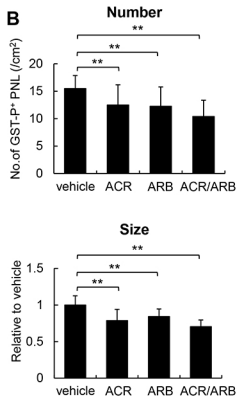

Supplement: Supplementary file 2 — Figure S1. Effects of acyclic retinoid (ACR) and angiotensin-II receptor blocker (ARB) on hepatocarcinogenesis in non-diabetic rats. (A) Representative microphotographs of glutathione S-transferase (GST-P)-positive preneoplastic foci. (B) Number of GST-P positive neoplastic lesions (GST-P+ PNL) per square centimeter (Upper panel) and relative size of GST-P+ PNL (Lower panel). LETO; Long-Evans Tokushima Otsuka rat, Data are mean ± SD (n = 10). **, P ≤ 0.05 between each group (B). (PDF 927 kb) [file 12885_2018_5099_MOESM2_ESM.pdf]

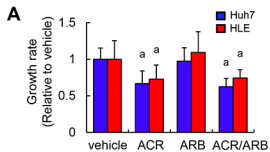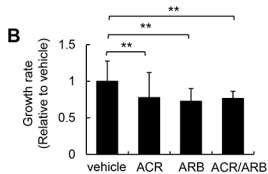

Supplement: Supplementary file 3 — Figure S2. Effects of acyclic retinoid (ACR) and angiotensin-II receptor blocker (ARB) on liver cancer cell and human umbilical vein endothelial cell (HUVEC) growth cultured under normal condition.(A) The effects of ACR and/or ARB on the proliferation of human liver cancer lines cultured under condition. (B) The effects of ACR and/or ARB on the proliferation of HUVECs cultured under condition. Data are mean ± SD (n = 10). a, P ≤ 0.01 as compared with the vehicle-mediated group (A); **, P ≤ 0.05 between each group (B). (PDF 211 kb) [file 12885_2018_5099_MOESM3_ESM.pdf]
